# Long-Term Outcomes of Indirect Annuloplasty for Functional Mitral Regurgitation: Interim Results From the CINCH Registry

**DOI:** 10.1016/j.shj.2025.100738

**Published:** 2025-10-12

**Authors:** Stephan Fichtlscherer, Sergiu Hicea, Fabian Barbieri, Murat Yildiz, Elvis Ypi, Humam Al-Kadah, Dietrich Pfeiffer, Steven L. Goldberg, Klaus K. Witte, Horst Sievert, CINCH Investigators

**Affiliations:** a Segeberger Kliniken: Herz und Gefäßzentrum, Segeberg, Germany; b Herz und Gefäßzentrum, Universitätsklinik der Goethe Universität, Frankfurt, Germany; c Klinikum Westmünsterland, St. Marien-Krankenhaus, Ahaus, Germany; d Deutsches Herzzentrum der Charité, Department of Cardiology, Angiology and Intensive Care, Campus Benjamin Franklin, Berlin, Germany; e Charité, Universitaetsmedizin Berlin, Freie Universitaet Berlin and Humbolt University Berlin, Department of Cardiology, Berlin, Germany; f Institute of Active Polymers and Berlin Brandenburg Center for Regenerative Therapies, Helmholz-Zentrum Hereon, Tetlow, Germany; g Klinik für Kardiologie, Elisabeth-Krankenhaus, Kassel, Germany; h Klinik für Kardiologie und Pulmologie, Hospital Zum Heiligen Geist, Fritzlar, Germany; i Klinik für Kardiologie, Universitätsklinik Leipzig AöR, Leipzig, Germany; j Cardiac Dimensions, Kirkland, Washington, USA; k Leeds Institute for Cardiometabolic Medicine, University of Leeds and Leeds Teaching Hospitals NHS Trust, Leeds, UK; l CVC Rhein Main, Frankfurt, Germany

**Keywords:** Carillon Mitral Contour System, Functional mitral regurgitation, Heart failure, Indirect percutaneous mitral annuloplasty

## Abstract

**Background:**

Functional mitral regurgitation (FMR) has limited effective treatment options. As a transcatheter indirect annuloplasty, the Carillon Mitral Contour System reduces FMR and improves symptoms, but long-term safety and effectiveness are incompletely characterized.

**Methods:**

This prospective, multicenter CINCH registry enrolled heart failure patients with FMR treated with the Carillon Mitral Contour System across 22 centers in Germany. Main outcomes included change in New York Heart Association (NYHA) class and MR severity, heart failure hospitalization (HFH), all-cause mortality rates, and device- or procedure-related serious adverse events at 1 year. Median follow-up was 2.0 years (range, 0-5 years).

**Results:**

Among 228 patients (age 78 ± 8 years; 51% female; 51% with left ventricular ejection fraction ≥50%), Carillon implantation improved clinical and echocardiographic variables. The rate of NYHA class III or IV symptoms decreased from 81 to 29% at 1 year, and the rate of moderate-to-severe MR (grade 3+/4+) decreased from 84 to 9% at 1 year, with ≥92% showing stable or improved NYHA class and ≥97% showing stable or improved MR grade over 5 years. Decreases in MR grade were associated with improvements in NYHA class (*p* = 0.048). Kaplan–Meier estimates over 5 years were 54.0% for HFH, 46.9% for all-cause mortality, and 68.3% for the composite of HFH or death. The proportion of patients experiencing any serious device- or procedure-related serious adverse event at 1 year was 1.8%.

**Conclusions:**

The CINCH registry provides real-world evidence supporting the long-term safety, effectiveness, and durability of the Carillon Mitral Contour System in treating FMR.

## Introduction

Functional mitral regurgitation (FMR), defined as mitral regurgitation in the absence of organic mitral valve disease, represents a significant cardiovascular burden, occurring in up to 50% of patients with heart failure (HF), with a direct relationship between severity and mortality risk.^1^ Although guideline-directed medical therapy (GDMT) is the first-line treatment approach for HF, improvements in MR severity are unpredictable,^2^ not least due to limited medical options in HF with preserved ejection fraction (HFpEF). Although resistant MR augurs a particularly poor prognosis across all categories of HF,^3^ surgical approaches are applicable to very few patients due to overwhelming operative risks in this elderly and multimorbid population,^4^ prompting the development of alternative transcatheter approaches.

Transcatheter edge-to-edge repair (TEER), a percutaneous procedure that creates a double-orifice mitral valve by clipping the leaflets together, is one treatment option for FMR. Guideline indications are based upon 3 landmark trials,^5^, ^6^, ^7^ with somewhat divergent results. While the COAPT (Cardiovascular Outcomes Assessment of the MitraClip Percutaneous Therapy for Heart Failure Patients with Functional Mitral Regurgitation) trial demonstrated that TEER can reduce heart failure hospitalization (HFH) and all-cause mortality, the RESHAPE-HF2 (Randomized Study of the MitraClip Device in Heart Failure Patients With Clinically Significant Functional Mitral Regurgitation) trial showed an effect on HF hospitalizations but no differences in mortality, and the MITRA-FR (Percutaneous Repair with the MitraClip Device for Severe Functional/Secondary Mitral Regurgitation) trial showed no difference between TEER and standard medical therapy on any patient-orientated outcome. Subsequent analyses have proposed that TEER may be less beneficial in patients with severe left ventricular (LV) dilation or dysfunction with compromised leaflet coaptation.^8^ These anatomical features, representing markers of lesser response, have contributed to the restricted indications for TEER to patients with “COAPT-like” features.

The Carillon Mitral Contour System is an alternative transcatheter approach that uses indirect annuloplasty via the coronary sinus to reduce septal-lateral annular dimensions and thereby improve leaflet coaptation. In several prospective trials, including the randomized sham-controlled REDUCE-FMR (Carillon Mitral Contour System for Reducing Functional Mitral Regurgitation) trial, the device significantly reduced regurgitant volume, induced reverse LV remodeling, and improved functional capacity.^9^^,^^10^ These trials employed strict inclusion criteria by enrolling patients with reduced LV ejection fraction (LVEF ≤40% in TITAN [Treatment of functional mitral regurgitation by percutaneous annuloplasty] and TITAN II^11^^,^^12^; ≤50% in REDUCE-FMR^13^). Consequently, the generalizability of these findings to unselected patients encountered in routine clinical practice warrants additional investigation. The CINCH postmarket registry was established to address this knowledge gap by evaluating the long-term outcomes of Carillon therapy in up to 500 patients with HF and FMR treated in routine clinical practice. The short-term clinical outcomes of the first 101 patients enrolled in the registry have been previously reported.^14^ We now report updated results from the first 228 patients treated in the prospective multicenter CINCH registry, with follow-up available for up to 5 years.

## Methods

### Study Design and Population

The CINCH FMR registry is a prospective, multicenter, single-arm, postmarket study evaluating the Carillon Mitral Contour System (Cardiac Dimensions, Pty Ltd, Kirkland, Washington, United States) in patients with symptomatic HF and FMR treated across 22 centers in Germany. Where applicable, multidisciplinary Heart Teams at each institution independently made treatment decisions according to local practice patterns but based on the European Society of Cardiology guidelines for the diagnosis of secondary mitral regurgitation.^15^ The inclusion criteria were minimal to maximize generalizability to routine clinical practice, requiring only age ≥18 years and a clinical diagnosis of FMR made by the Heart Team at the treating center. Apart from the on-label indications for the device, enrollment into the registry did not require a particular degree of MR severity, ventricular dimensions, LVEF, or HF severity.

The study was prospectively registered at www.clinicaltrials.gov (NCT05677568), the ethics committees at each participating center approved the protocol, all participants provided written informed consent, and study procedures adhered to the Declaration of Helsinki.

### Procedure

The Carillon implantation technique has been described in detail previously.^10^ Under conscious sedation or general anesthesia, the 9F delivery system is advanced through the right internal jugular vein to the coronary sinus. The Carillon device, a fixed-length nitinol bridge with proximal and distal anchors, is then positioned to span the posterior mitral annular circumference. After confirming correct positioning, the distal anchor is deployed in the great cardiac vein, and controlled tension is then applied by retracting the delivery system to reduce the septal-lateral dimension of the mitral annulus. Once the planned retraction has been achieved, the proximal anchor is deployed near the coronary sinus ostium to maintain tension. Before final release, anchor stability and absence of clinically significant compression of the circumflex artery or other adjacent coronary vessels are confirmed. Postprocedural management includes the continuation (or titration) of GDMT.

### Follow-Up and Data Collection

The registry implemented a pragmatic follow-up schedule aligned with standard clinical practice at participating institutions. The recommended assessment intervals included evaluations at 1 month, 6 months, 1 year, and annually thereafter until 5 years postimplantation. Each follow-up visit included a clinical assessment for New York Heart Association (NYHA) functional classification and documentation of adverse events and HFH. Transthoracic echocardiography was performed at each visit, and MR severity was graded using standardized criteria.^16^ LVEF was quantified at each echocardiographic assessment using the biplane Simpson's method when feasible, or by visual estimation when the endocardial definition was suboptimal.

### Endpoints and Definitions

The primary outcomes were changes in NYHA class, changes in MR severity, all-cause mortality, and HFH. HFH was defined as any hospital admission with a primary diagnosis of decompensated HF requiring intravenous diuretic or vasoactive therapy, with a minimum duration of 24 hours or calendar date change. Safety outcomes included device-related serious adverse events up to 1 year after implantation, defined as complications attributable to the device, such as anchor dislocation or embolization, device fracture, coronary sinus perforation or dissection, or myocardial ischemia resulting from coronary artery compression. Procedure-related serious adverse events were defined as complications related to the implantation procedure, such as vascular access complications, anesthesia-related events, periprocedural arrhythmia, and contrast-induced nephropathy.

### Statistical Analysis

Baseline characteristics are presented as means and SDs for continuous variables and frequencies and percentages for categorical variables. Changes in NYHA class, MR grade, and LVEF from baseline to each follow-up interval were analyzed using paired samples *t*-tests. Fisher exact test was used to compare NYHA class improvement between patients with and without MR grade reduction. Time-to-event outcomes (HFH, mortality) were analyzed using Kaplan–Meier methods. All statistical tests were two-sided, with a threshold for statistical significance set at *p* < 0.05. Statistical analyses were performed using Stata v19.5 (StataCorp).

## Results

A total of 228 consecutive patients were treated with the Carillon Mitral Contour System at 22 centers in Germany between December 2012 and October 2024. The mean age of this cohort was 78 ± 8 years, 51% were female, and most (70%) had NYHA class III symptoms (12% with class IV). Most patients had moderate-to-severe MR (84% with MR grade 3+ or 4+). The mean LVEF was 46% ± 14%, and 51% of patients had an LVEF ≥50%. Overall, the cohort had a high prevalence of cardiovascular comorbidities, including hypertension (83%), atrial fibrillation (75%), and coronary artery disease (57%) (Table 1). Adherence to GDMT was high, with 83% of patients taking beta-blockers, 83% taking diuretics, 78% taking angiotensin converting enzyme inhibitors/angiotensin receptor blockers or angiotensin receptor neprolysin inhibitors, and 47% taking mineralocorticoid receptor antagonists. Given the period covered, few were taking sodium-glucose transport 2 inhibitors.

**Table 1:** Characteristics of participants in the CINCH cohort study

Characteristic	Total (n = 228)	HFrEF (n = 103)	HFpEF (n = 108)	*p* value
Demographics				
Age (y)	78 ± 8	75 ± 9	80 ± 6	<0.001
Male sex	49% (112/228)	62% (64/103)	35% (38/108)	<0.001
Medical history				
Hypertension	83% (190/228)	79% (81/103)	89% (96/108)	0.06
Atrial fibrillation	75% (170/228)	64% (66/103)	86% (93/108)	<0.001
Coronary artery disease	57% (129/228)	62% (64/103)	48% (52/108)	0.05
Ischemic etiology	47% (106/225)	62% (63/102)	32% (34/107)	<0.001
Myocardial infarction	23% (52/228)	32% (33/103)	13% (14/108)	<0.001
ICD	12% (28/228)	21% (22/103)	5% (5/108)	<0.001
Clinical status				
NYHA classification				0.25
I	1% (2/223)	2% (2/101)	0% (0/105)	
II	18% (40/223)	15% (15/101)	23% (24/105)	
III	70% (155/223)	70% (71/101)	66% (69/105)	
IV	12% (26/223)	13% (13/101)	11% (12/105)	
Left ventricular variables				
LV ejection fraction (%)	46 ± 14	34 ± 9	58 ± 6	<0.001
Mitral valve variables				
MR grade (at rest)				0.07
1	<1% (1/225)	1% (1/103)	0% (0/107)	
2	16% (35/225)	10% (10/103)	21% (23/107)	
3	57% (129/225)	63% (65/103)	55% (59/107)	
4	27% (60/225)	26% (27/103)	23% (25/107)	

*p* values were calculated using independent samples *t*-tests for continuous data and Fisher exact test for categorical data. Values are presented as mean ± SD for continuous variables and percentage (n/N) for categorical data.

Abbreviations: HFpEF, heart failure with preserved ejection fraction; HFrEF, heart failure with reduced ejection fraction; ICD, implantable cardioverter-defibrillator; LV, left ventricular; MR, mitral regurgitation; NYHA, New York Heart Association.

**Figure 1:**
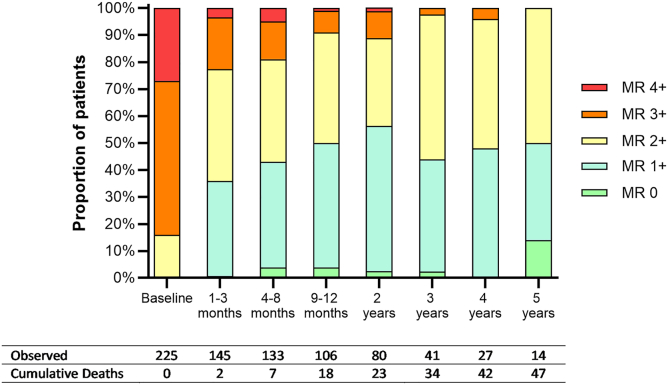
**Changes in distribution of MR classification following indirect annuloplasty using the Carillon device** ∗Values are presented as percentage. Abbreviation: MR, mitral regurgitation.

**Figure 2:**
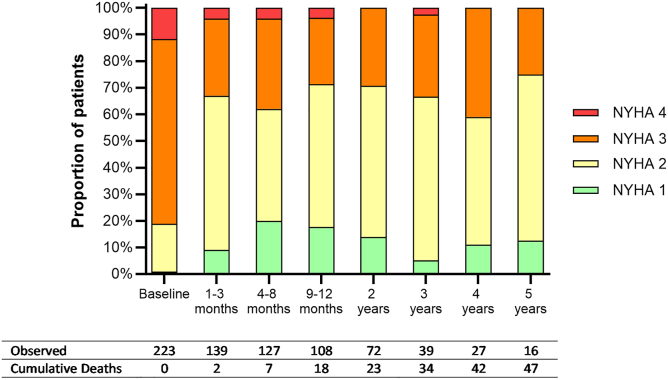
**Change in distribution of NYHA classification following indirect annuloplasty using the Carillon device** ∗Values are presented as percentage. Abbreviation: NYHA, New York Heart Association.

Patients were followed up for a median of 2.0 years (range, 0-5 years). The severity of MR showed a consistent improvement over time. The proportion of patients with moderate-to-severe MR (grade 3+ or 4+) decreased from 84% at baseline to 9% at 1 year, and ranged between 0% and 11% thereafter (Figure 1). The MR grade improved or remained stable in ≥97% of patients throughout the follow-up period, and the overall mean decrease in the MR grade was statistically significant at all follow-up intervals (all *p* < 0.001). The proportion of patients with NYHA class III or IV symptoms also decreased from 81% at baseline to 29% at 1 year and ranged from 25% to 41% thereafter (Figure 2). NYHA classification improved or remained stable in ≥92% of patients across all follow-up intervals, and the overall mean decrease in NYHA class was statistically significant at all follow-up intervals (all *p* < 0.001). Reductions in MR grade were associated with improvements in NYHA class. Individuals in whom there was a *reduction* in MR grade were more likely to experience an *improvement* in NYHA class (72.2 vs 41.7% [*p* = 0.048]).

**Figure 3:**
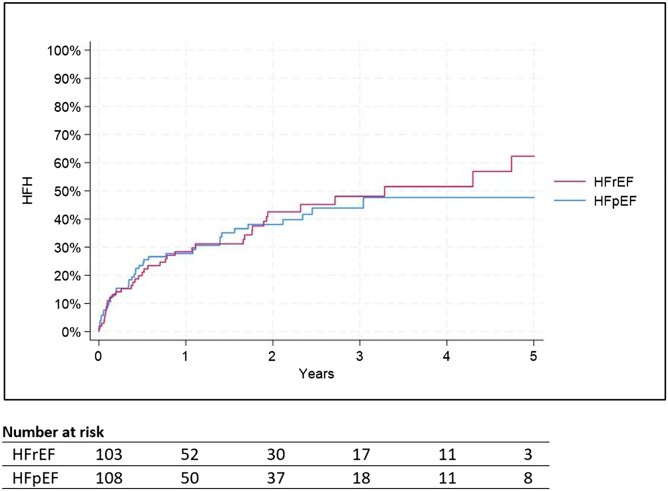
**Time to HFH over 5 years following indirect annuloplasty using the Carillon device** Log rank *p* value <0.001 for group comparison. Standard errors remained below 10% in each group throughout the follow-up period. Abbreviations: HFH, heart failure hospitalization; HFpEF, heart failure with preserved ejection fraction; HFrEF, heart failure with reduced ejection fraction.

**Figure 4:**
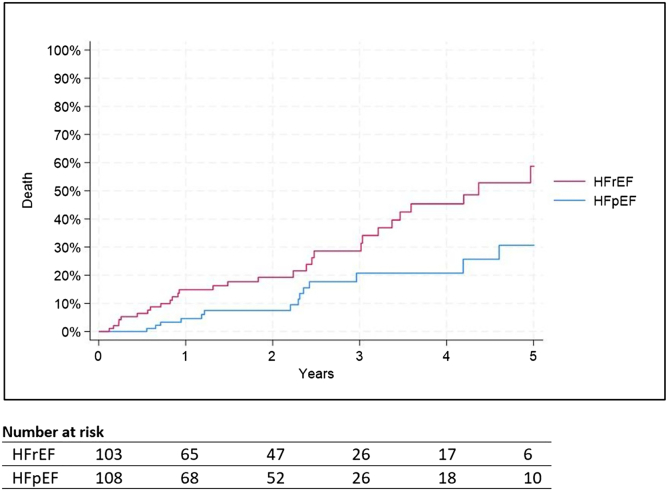
**Time to death over 5 years following indirect annuloplasty using the Carillon device** Log rank *p* value <0.001 for group comparison. Standard errors remained below 10% in each group throughout the follow-up period. Abbreviations: . HFrEF, heart failure with reduced ejection fraction; HFpEF, heart failure with preserved ejection fraction.

**Figure 5:**
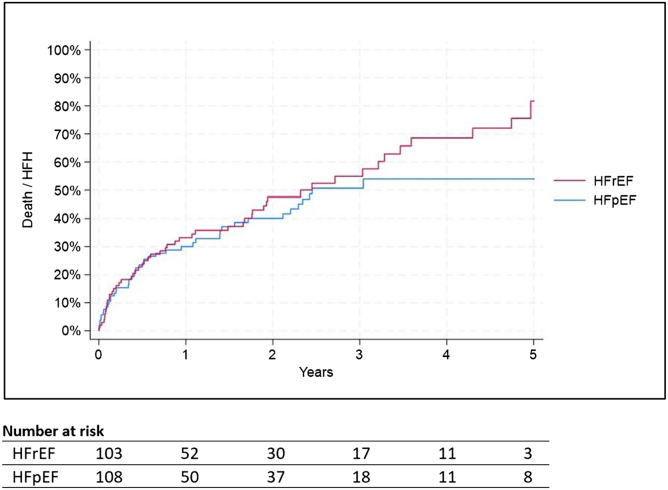
**Time to HFH or death over 5 years following indirect annuloplasty using the Carillon device** Log rank *p* value <0.001 for group comparison. Standard errors remained below 10% in each group throughout the follow-up period. Abbreviations: HFH, heart failure hospitalization; HFpEF, heart failure with preserved ejection fraction; HFrEF, heart failure with reduced ejection fraction.

Kaplan–Meier estimates for HFH were 26.1% at 1 year and 54.0% at 5 years (Figure 3). The corresponding all-cause mortality rates were 9.4 and 46.9%, respectively (Figure 4). The composite endpoint of HFH or death occurred in 29.8% of patients at 1 year and 68.3% at 5 years (Figure 5). These rates were different for people with HF with reduced ejection fraction versus HFpEF (58.8 vs 30.7%; *p* < 0.001 for mortality, 62.3 vs 47.7%; *p* < 0.001 for HFH, and 81.7 vs 54.0%; *p* < 0.001 for the composite endpoint).

The Carillon device demonstrated a favorable safety profile with no device or procedure-related 30-day mortality. At 1 year, 4/228 (1.8%) patients experienced device- or procedure-related serious adverse events. These events included 2 cases of persistent severe mitral insufficiency, one case of non-ST-elevation myocardial infarction, and one case of pericardial effusion, which was classified as both device- and procedure-related, representing the only procedure-related serious adverse event (0.4%).

## Discussion

This multicenter postmarket registry demonstrates that the Carillon Mitral Contour System provides sustained clinical and hemodynamic benefits in an unselected real-world FMR population. Our findings demonstrated significant and durable improvements in HF symptoms and in the proportion of patients with moderate-to-severe or severe MR with the structural changes related to the improvements in symptoms. These benefits were achieved with an excellent safety profile, with only 1.8% experiencing a device- or procedure-related serious adverse event. The 5-year composite endpoint of death or HFH occurred in 68.3% of patients, representing a reasonable long-term outcome in this elderly cohort with advanced HF symptoms and extensive comorbidities, and approximating the rates reported in previous Carillon clinical trials.^17^ Collectively, these findings substantiate the durability of indirect annuloplasty with the Carillon system and establish its potential utility across a broader spectrum of FMR patients than in previous trials.

These results are particularly noteworthy for patients with preserved LVEF, as these populations were excluded from previous Carillon trials. The inclusion of these understudied subgroups addresses an important knowledge gap and points at a broader therapeutic potential for transcatheter indirect annuloplasty than previously established.

Several limitations of this study warrant consideration. First, despite data collection procedures, there were 5 patients in whom NYHA information was not available. Secondly, the absence of a randomized control group precludes the definitive attribution of clinical improvements to the device rather than natural history or regression to the mean, or additional medical therapy. Moreover, we have not undertaken a comparison with TEER or surgical registries since this population would compare poorly with either COAPT-like patients or those with atrial FMR deemed suitable for TEER due to differing remodeling. Given the age of the present population, comparison with a surgical registry would be even more unhelpful. The ongoing EMPOWER [Assessment of the Carillon Mitral Contour System in Treating Heart Failure with Functional Mitral Regurgitation] randomized trial comparing Carillon therapy to sham therapy (www.clinicaltrials.gov; NCT05677568) will provide more definitive evidence. Third, site-performed echocardiographic assessments without central core laboratory adjudication may introduce outcome variability, although this approach reflects real-world clinical practice. Moreover, as a result of this approach, we had limited data on left atrial size and other clinical variables which may modify the mechanistic or symptomatic responses to this coronary sinus-based therapy.^18^^,^^19^ Such analyses will require larger and more detailed data sets, which are not available, but the currently enrolling EMPOWER study will allow for this type of analysis. Fourth, survivor bias may have influenced the long-term outcomes, as patients with longer follow-up represent a potentially healthier subset who survived and remained engaged in care. Fifth, although medication use was documented at baseline, systematic tracking during follow-up was not mandated, which obscured the relative contributions of medical therapy versus the device effect. Sixth, quality of life metrics and objective exercise capacity measures were not systematically collected, which limited the ability to quantify functional improvement beyond NYHA classification. Finally, despite the broad inclusion criteria to improve generalizability, the study was conducted exclusively at experienced centers in Germany, which may limit its applicability to lower-volume institutions or health care systems with different patient demographics and treatment patterns.

## Conclusions

The CINCH registry demonstrates, in a real-world environment, that the Carillon device leads to a sustained reduction in MR severity, stable benefits on functional status over 5 years of follow-up, and an excellent safety profile in a wide range of patients, including those with HFpEF.

## Ethical Statement

Although enrollment was consecutive and unselected according to usual pathways of care, each institution achieved local ethical approval and each participant provided informed, written consent for the collection of their data. The study was conducted according to the Declaration of Helsinki.

## Funding

The CINCH registry was sponsored by Cardiac Dimensions, Pty Ltd. (Kirkland, WA, United States).

## Disclosure Statement

Stephan Fichtlscherer reports study honoraria to institution, travel expenses, consulting fees from Abbott, Amgen, Edwards, Cardiac Dimensions, Medtronic, Boston Scientific, Biosensors, Novartis, Abbott, BMS, Pfizer, Bayer, and Siemens Healtheneers. Fabian Barbieri reports grant support from Abbott Laboratories and Boston Scientific consulting fees from Boston Scientific and Edwards Lifesciences and speaker honoraria from Edwards Lifesciences. Steven L. Goldberg is a consultant for Cardiac Dimensions and Abbott. Klaus K. Witte has received personal fees for educational activities from Medtronic, Cardiac Dimensions, Novartis, Abbott, BMS, Pfizer, and Bayer; and has received an unconditional research grant from Medtronic. Horst Sievert reports study honoraria to institution, travel expenses, consulting fees from Abbott, Ablative Solutions, Adona Medical, Akura Medical, Ancora Heart, Append Medical, Axon, Bavaria Medizin Technologie GmbH, BioRefine, Boston Scientific, Canon, Cardiac Dimensions, Cardiac Success, Cardimed, Cardionovum, Contego, Coramaze, Croivalve, CSL Behring LLC, CVRx, Dinova, Eclipse, Endobar, Endologix, Endomatic, Esperion Therapeutics, Inc, Hangzhou Nuomao Medtech, Holistick Medical, Intershunt, Intervene, Laminar, Lifetech, Magenta, Maquet Getinge Group, Metavention, Mitralix, Mokita, Myotec, Neurotronic, Novelrad, NXT Biomedical, Occlutech, Recor, Renal Guard, Shifamed, Terumo, Tonic Medical, Trilio, Trisol, TruLeaf, Vascular Dynamics, Vectorious Medtech, Venus, Venock, Vivasure Medical, Vvital Biomed, Whiteswell, and Xenter. The other authors had no conflicts to declare.
